# Improving Sepsis Prediction in the ICU with Explainable Artificial Intelligence: The Promise of Bayesian Networks

**DOI:** 10.3390/jcm14186463

**Published:** 2025-09-13

**Authors:** Geoffray Agard, Christophe Roman, Christophe Guervilly, Mustapha Ouladsine, Laurent Boyer, Sami Hraiech

**Affiliations:** 1 Service de Médecine Intensive—Réanimation, AP-HM, Hôpital Nord, 13015 Marseille, France; 2 Centre d’Etudes et de Recherches sur les Services de Santé et qualité de vie (CERESS) EA 3279, Faculté de Médecine, Aix-Marseille Université, 13005 Marseille, France; 3 Laboratoire d’Informatique et Système (LIS) UMR 7020 CNRS/AMU/UTLN, Aix Marseille Université—Campus de Saint Jérôme—Bat. Polytech, 13013 Marseille, France; 4 Département d’Information Médicale, AP-HM, Hôpital de la Conception, 13005 Marseille, France

**Keywords:** Artificial Intelligence (AI), Bayesian networks, explainable AI (xAI), sepsis, intensive care unit (ICU), prediction, CDSS

## Abstract

**Background/Objectives:** Sepsis remains one of the leading causes of mortality worldwide, characterized by a complex and heterogeneous clinical presentation. Despite advances in patient monitoring and biomarkers, early detection of sepsis in the intensive care unit (ICU) is often hampered by incomplete data and diagnostic uncertainty. In recent years, machine learning models have been proposed as predictive tools, but many function as opaque “black boxes”, meaning that humans are unable to understand algorithmic reasoning, poorly suited to the uncertainty-laden clinical environment of critical care. Even when post-hoc interpretability methods are available for these algorithms, their explanations often remain difficult for non-expert clinicians to understand. **Methods:** In this clinical perspective, we explore the specific advantages of probabilistic graphical models, particularly Bayesian Networks (BNs) and their dynamic counterparts (DBNs), for sepsis prediction. **Results:** Recent applications of AI models in sepsis prediction have demonstrated encouraging results, such as DBNs achieving an AUROC of 0.94 in early detection, or causal probabilistic models in hospital admissions (AUROC 0.95). These models explicitly represent clinical reasoning under uncertainty, handle missing data natively, and offer interpretable, transparent decision paths. Drawing on recent studies, including real-time sepsis alert systems and treatment-effect modeling, we highlight concrete clinical applications and their current limitations. **Conclusions:** We argue that BNs present a great opportunity to bridge the gap between artificial intelligence and bedside care through human-in-the-loop collaboration, transparent inference, and integration into clinical information systems. As critical care continues to move toward data-driven decision-making, Bayesian models may offer not only technical performance but also the epistemic humility needed to support clinicians facing uncertain, high-stakes decisions.

## 1. Introduction

Sepsis remains one of the leading causes of mortality worldwide, with an estimated 11 million deaths annually in 2017, accounting for 19.7% of all-cause mortality worldwide [1]. Since the 2016 consensus conference (Sepsis-3), it has been defined as an infectious episode leading to life-threatening organ dysfunction caused by an inappropriate host response to infection [2]. The pathophysiology of sepsis is complex and highly heterogeneous: it involves dysregulation of the immune response, microcirculatory disturbances, metabolic imbalances, and close interactions with the patient’s underlying condition like age, comorbidities or immunosuppression. In addition to these biological factors, there is also variability in clinical presentation: some patients have a mild inflammatory response, while others experience sudden failure with no apparent signs of infection. In the intensive care unit (ICU), sepsis management mobilizes significant resources and requires clinicians to make rapid decisions in a context of high uncertainty. Intensivists are often confronted in their practice with mild, early, or atypical forms of sepsis, which are common particularly in ventilator-associated pneumonia or catheter-related infections. Despite their widespread use, currently available biomarkers such as C-reactive protein, procalcitonin, and lactate suffer from limited diagnostic performance. Combined with the lack of an objective gold standard, this continues to hinder the timely, reliable, and clinically actionable identification of sepsis, ultimately limiting our ability to significantly improve patient outcomes. Similarly, therapeutic decisions such as the introduction of antibiotic therapy, the use of vasopressors, or the indication for invasive monitoring are generally based on clinical reasoning that must account for incomplete, uncertain, or ambiguous signals. In the ICU, clinicians are continuously confronted with a dense and heterogeneous flow of information, ranging from bedside monitors to laboratory results, imaging studies, and clinical documentation. While this abundance of data has the potential to improve decision-making, it often generates cognitive overload, fragmenting attention and making it difficult to identify truly relevant patterns. As a result, the ability to integrate signals into a coherent clinical picture is impaired, which may compromise nuanced judgment and reflective medical reasoning.

This complexity has led to growing interest in artificial intelligence (AI) techniques within the medical community. The appealing idea behind such tools enabling early diagnosis before the warning signs become obvious led us to consider optimizing one of the key elements of treatment: the fastest administration of appropriate antibiotic therapy after diagnosis. While the link between patient prognosis and a prompt and appropriate antibiotic administration is well established in sepsis [3,4,5,6], data on the potential benefits of AI tools for improving key clinical outcomes, such as mortality or hospital stay duration, remain scarce. Some studies have already shown that the implementation of AI-based early sepsis alert systems significantly improves patient outcomes [7]. However, in practice, many of these models are little more than black boxes that are difficult to reconcile with clinical requirements. They require complete data to operate, reject ambiguity, and struggle to explain their recommendations. These limitations significantly hinder their integration into real-world clinical workflows, where uncertainty is the rule rather than the exception.

This is precisely what led us to explore Bayesian networks (BN), probabilistic models that are increasingly recognized for their ability to formalize clinical uncertainty, integrate expert knowledge, and generate transparent, interpretable inferences. Unlike many conventional machine learning models that attempt to “guess” an outcome from a set of inputs, Bayesian networks mimic the structure of clinical reasoning: they represent hypotheses that are continuously updated as new evidence becomes available. These models explicitly “acknowledge” their own epistemic limitations, can operate effectively even in the presence of incomplete information, and allow clinicians to understand the rationale behind each prediction. This intrinsic explainability, combined with their alignment with the reasoning processes used daily in critical care, positions BNs as particularly promising tools for bedside decision support in intensive care units.

This perspective aims to highlight the largely untapped potential of Bayesian probabilistic models in the field of sepsis prediction. To this end, we first provide an educational introduction to conditional probability models with a focus on BNs. We then critically review the main studies that have applied such models to sepsis care, across three clinical dimensions: early diagnostic support, patient stratification and prognosis, and therapeutic decision-making. Finally, we reflect on the current barriers and future directions required for the meaningful integration of these models into routine intensive care practice.

## 2. Why Are Traditional AI Models Struggling to Gain Acceptance in Intensive Care?

The field of artificial intelligence generally encompasses two distinct subfields: machine learning (ML), which focuses on statistical algorithms, and deep learning, which relies on structures based on deep neural networks [8]. Despite encouraging results, only 5% of models published in the literature are estimated to be tested in prospective studies to evaluate potential clinical adoption [9]. This mistrust can be explained by a well-known principle in data science: the “black box syndrome”. Some authors define the algorithmic black box as “any obscure data mining or machine learning model whose internal elements are either unknown to the observer or known but uninterpretable by humans” [10]. To take it simply, we can consider that black boxes can make accurate predictions, but the reasoning process behind these predictions remains hidden. This principle of human inability to understand the results produced by the AI system is one of the central elements in any adoption of new predictive medical technology. However, one of the main concerns for clinicians is to be able to partially trace the reasoning of the tool in order to retain their share of responsibility in the final decision [11].

These research areas are defined as eXplainable Artificial Intelligence (XAI), which aims to develop tools for deciphering the inference mechanisms of algorithms. There are a number of well-documented and effective explainability frameworks in ML (SHAP, LIME, etc.). For example, SHAP was inspired by game theory and assign a “score” to each variable to show how much it contributed to a given prediction. Although some prediction explainability techniques have recently emerged for DL (e.g., integrated gradient technique, attention mechanisms in transformers) [12,13], their use remains fairly limited in literature. However, this is not a sufficient reason to avoid using the tools currently available, some of which have already shown spectacular results in improving clinical outcomes. The most striking example is the use of the TREWS (Targeted Real-time Early Warning System) algorithm in a prospective multicenter trial [14]. The purpose of this tool was to alert clinicians in real time if the 3 h future risk of sepsis was high. If the clinician considered the alert to be justified, they would begin antibiotic treatment. In the group using the algorithm, an absolute reduction in the risk of in-hospital death of 18% was observed, highlighting the major impact that these technologies can have on patient prognosis [7]. Despite this great success, algorithms such as TREWS have certain drawbacks. They cannot function in the presence of missing data (which is commonplace in our clinical practice), they are not fully explainable, and they determine a diagnosis irrevocably without expressing any uncertainty. The major limitation of these explainability methods is their post-hoc nature: we try to understand, after the prediction has been made, why the algorithm produced a given result. Such explanations may vary depending on data sampling, leading to inconsistent justifications from one patient to another. In addition, the outputs of these tools are sometimes difficult to interpret clinically (for example, a SHAP value of 0.12 for the variable “age”). In daily practice, clinicians require models that are explicitly interpretable (for example, “age above 65 years increases the risk of death”), whose explanations remain stable and global, while at the same time quantifying uncertainty.

What if clinical decision support could rely on a model that not only quantifies its own predictive uncertainty, but also remains robust in the face of missing data and offers full mathematical and graphical interpretability? This is precisely what BNs offer. By directly addressing the core limitations that hinder the implementation of artificial intelligence at the bedside, namely opacity, inflexibility, and the inability to handle incomplete information, Bayesian networks represent a promising path toward clinically acceptable and actionable AI.

## 3. Conditional Probability Models: Principles and Foundations

### 3.1. Definition

Bayesian networks (BNs), also known as Bayesian belief networks, are probabilistic mathematical models that use a directed acyclic graph (DAG) to represent a set of variables and their conditional dependencies [15]. Introduced in the 1980s as a formalism for reasoning with uncertainty (notably by Judea Pearl [16]), BNs have since been successfully applied in many fields (environmental sciences, finance, genomics, etc.). In medicine, they are used to improve diagnosis and prognosis by enabling clinicians to reason probabilistically and integrate medical knowledge into formal models. In mathematical terms, BNs encode the joint distribution of all variables by factoring it according to the structure of the graph, applying Bayes’ theorem and the chain rule of conditional probabilities. Bayes’ theorem allows us to update a belief (or probability) about an event or latent state in light of new information. It states that:

$$P\left(H|D\right)=\frac{P\left(D|H\right)\times P\left(H\right)}{P\left(D\right)}$$

where:

- *H*: a hypothesis (e.g., “the patient has sepsis”),
- *D*: observed data (e.g., “elevated lactate levels”),
- *P*(*H*): the prior probability of the hypothesis (without knowledge of the test),
- *P*(*D*∣*H*): the likelihood, i.e., the probability of observing *D* if *H* is true,
- *P*(*D*): the total probability of observing *D* (all causes combined),
- *P*(*H*∣*D*): the posterior probability, i.e., the revised belief about *H* after seeing *D*.

From a medical standpoint, this formula accurately describes the reasoning process occurring in a clinician’s thoughts: They begin with a pre-test probability (e.g., “15% of patients in intensive care have sepsis upon admission”), then observe a piece of data D (e.g., “the patient has a temperature > 39 °C”). The clinician is aware of the likelihood associated with this data if the patient were indeed suffering from sepsis (e.g., 70% of septic patients have a fever), and updates their initial belief to obtain a post-test probability. In this context, BNs can be used in two ways: either as a descriptive model of relationships (e.g., a causal model of the pathophysiology of a disease) or as a predictive model to estimate the probability of an event (diagnosis, risk) based on certain observations.

### 3.2. Bayesian Network Structure

**Figure 1:**
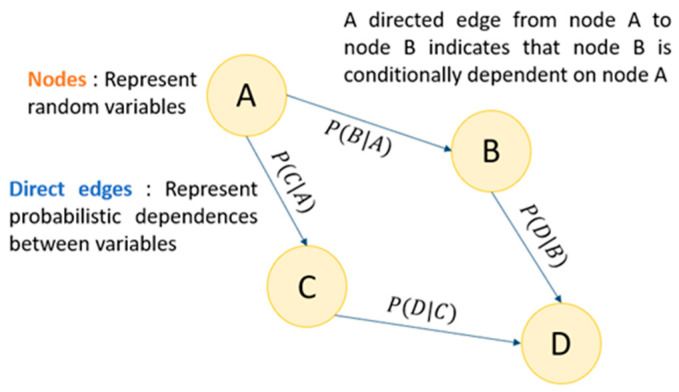
Bayesian Network representation with Directed Acyclic Graph.

The central element of a BN is its structural design based on a directed acyclic graph (DAG), where each node represents a variable (e.g., presence of fever, high lactate levels, sepsis, etc.) and each directed edge (arrow) indicates a conditional dependency relationship between variables (Figure 1). In practice, each node is associated with a conditional probability table (CPT) that specifies the probability of each possible state of the variable based on the states of its parents. These tables can be learned automatically from data or defined by medical experts based on available pathophysiological knowledge.

**Figure 2:**
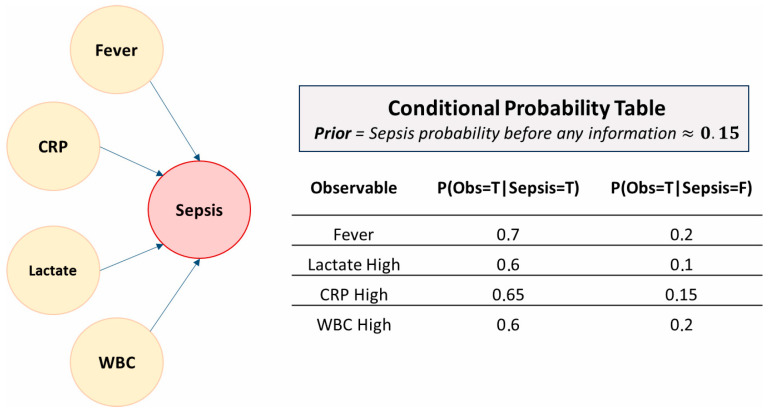
Illustrative Bayesian network for sepsis prediction. This synthetic BN models the probability of sepsis (red node) as a function of four observable clinical variables: fever, lactate level, C-reactive protein (CRP), and white blood cell count (WBC) (yellow nodes). Each arrow represents a probabilistic dependency, and the associated conditional probability table (CPT) specifies the likelihood of observing each clinical sign depending on whether sepsis is present (T) or absent (F). The prior probability of sepsis (≈15%) reflects the baseline risk before any patient-specific data are introduced.

Suppose we are treating a patient suspected of having sepsis. We seek to estimate the probability of this diagnosis based on 4 observable elements: Fever, C-Reactive Protein (CRP), blood lactate level and white blood cell count (WBC). We could have the following CPT (Figure 2):

One of the strengths of BNs is their ability to make probabilistic inferences about variables of interest based on partial evidence (e.g., biological test results, symptoms, etc.) through a process called belief propagation. This principle allows the algorithm to continue functioning in the presence of missing values, updating itself based on the new incomplete data it receives, just as a doctor would.

**Figure 3:**
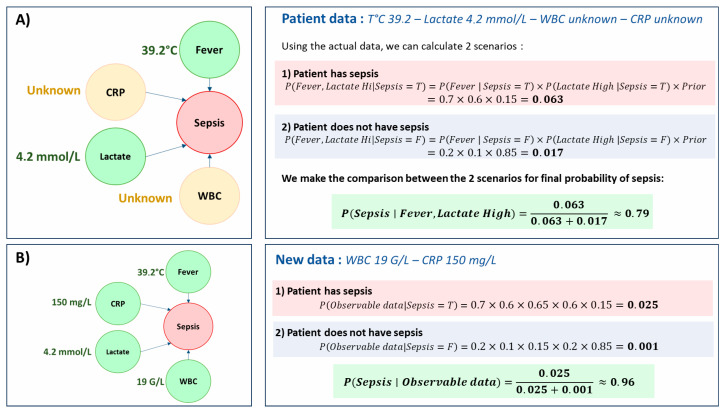
**Stepwise Bayesian inference for sepsis prediction with incomplete and updated data.** Panel (**A**) shows an initial scenario where the patient presents with fever (39.2 °C) and elevated lactate (4.2 mmol/L), while C-reactive protein (CRP) and white blood cell count (WBC) results are not yet available. Using these partial observations, the Bayesian network estimates a posterior probability of sepsis of approximately 79% using the conditional probability table given in Figure 2. Panel (**B**) illustrates how the probability is dynamically updated when new laboratory data arrive (WBC 19 G/L and CRP 150 mg/L). By integrating these additional findings, the network revises the posterior probability of sepsis upward to 96%.

For example, let us consider an intensivist managing a patient in the ICU for acute respiratory failure, likely secondary to pneumonia. The patient presents with a fever of 39.1 °C and a lactate level of 4.2 mmol/L. At this point, the white blood cell count, blood cultures, and C-reactive protein results are not yet available (Figure 3A). The blood pressure remains stable, the heart rate is moderately elevated, and the patient is slightly drowsy but not confused. In a traditional algorithm requiring a complete dataset, the clinician would have to wait for the remaining laboratory results, the completion of the medical record, and the full fulfillment of predefined criteria. However, in real-life clinical practice, decisions often need to be made rapidly to prevent further deterioration of the patient’s condition. This is where the BN comes into its own. It already “knows” that fever increases the likelihood of infection (by the training data). It also “knows” that hyperlactatemia, in a febrile context, points to a risk of sepsis. Even without the leukocytes count results, it is able to integrate these partial elements, assess the probabilities of potential causes, and produce an updated estimate of the risk of sepsis. If I receive additional information an hour later, such as an elevated CRP, the model updates immediately, without starting from scratch (Figure 3B). This example also illustrates a broader point: one of the main limitations of interpreting isolated clinical parameters is their lack of specificity. Leukocytosis, for instance, may indicate a new septic episode, but it can equally result from corticosteroid therapy or other non-infectious conditions. Rather than overinterpreting a single variable, the BN weighs the joint probability of multiple observations, which allows it to distinguish more reliably between sepsis and alternative explanations. This mechanism for belief propagation mirrors exactly the approach taken by clinicians: leveraging existing knowledge, integrating new evidence as it emerges, and continuously adjusting hypotheses. It is precisely this feature that makes these models so well-suited to the dynamic reality environment of the ICU.

### 3.3. Dynamic Bayesian Model (DBN) and Real Time Prediction

In the ICU, a patient’s condition is evolving very frequently. Vital signs vary from one hour to the next, biological tests accumulate, and therapeutic decisions alter the patient’s trajectory. Often, the decision is based not on the actual value at a given moment, but on how these values evolve over time; therefore, classical static BNs are not sufficient. This is where dynamic Bayesian networks (DBNs) come in to fill the gap.

**Figure 4:**
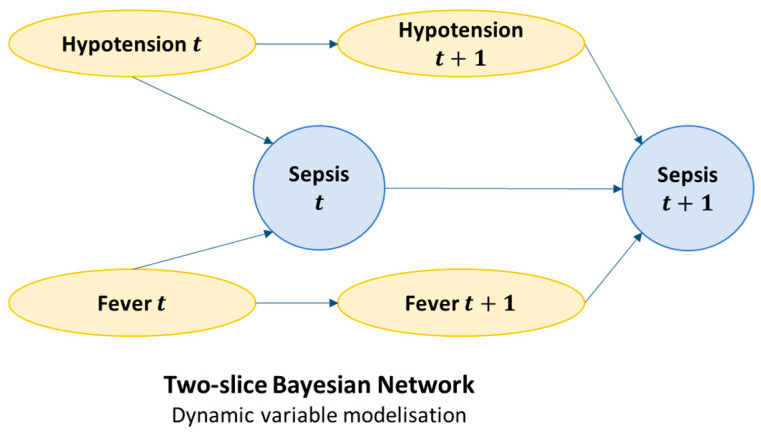
Representation of a two-slice Dynamic Bayesian Network (DBN).

A DBN is an extension of a static BN to time series. It allows us to model how variables change over time, taking into account both intra-temporal dependencies (within a given moment) and inter-temporal dependencies (between time point t and time point t + 1). A DBN is generally defined as a 2-Timeslice Bayesian Network (2TBN): a network that encodes the relationships between variables at two successive moments in time, then repeats this structure identically for each time step (Figure 4). Time t corresponds to the patient’s current state, and time t + 1 to their state in 6 h, 12 h, etc. This amounts to stacking a series of interconnected Bayesian networks linked by temporal arcs. As in classical Bayesian networks, inference can be performed: calculating the probability that the patient will have sepsis at t + 1 based on past observations [17].

In practical terms, consider an intensivist managing a patient undergoing weaning from mechanical ventilation after severe community-acquired pneumonia. On admission, the patient’s condition is stable: heart rate of 92 bpm, temperature of 37.6 °C, normal blood pressure, normal SpO_2_ with an FiO_2_ of 0.4. Lactate levels are 1.8 mmol/L, and C-reactive protein is elevated but trending downward. At this stage, the likelihood of sepsis appears low, or at least inactive. A few hours later, the clinician observes tachycardia at 110 bpm, mild hypotension at 95/55 mmHg, still no fever, but a subtle increase in confusion. Arterial blood gas analysis now shows lactate levels at 3.2 mmol/L. A static model would assess this situation in isolation, asking: “Does this combination of current variables correspond to sepsis?” In contrast, a DBN incorporates the temporal evolution of the patient’s status: it integrates prior information from earlier assessments, recognizes the concerning trajectory, and leverages its training data to identify this dynamic pattern as one frequently preceding sepsis onset. Such a model therefore evaluates not only the present clinical state, but also the patient’s progression to this point and the likely implications for the coming hours. The abundance of longitudinal data, such as time series, makes the use of DBNs particularly appealing in the ICU setting. Whereas most algorithms require regularly sampled inputs, DBNs can accommodate both missing values and variably sampled time series, which is a common situation for many clinical variables, particularly laboratory data.

In contrast to static BNs, DBNs also allow variables to be modeled as continuous rather than necessarily categorized or binarized. This approach helps to limit the loss of information that typically occurs when continuous variables are converted into discrete ones. Nevertheless, both approaches have their merits. Continuous modeling preserves the full richness of the data and allows the network to capture subtle variations (e.g., progressive increases in lactate or temperature). However, it requires stronger distributional assumptions (often conditional Gaussian models) and may reduce interpretability for clinicians who are accustomed to threshold-based reasoning [18]. Discretization, in turn, simplifies the conditional probability tables and aligns naturally with clinical practice, where decision-making is often guided by categorical cut-offs (lactate > 2 mmol/L, temperature > 38.3 °C). Its main limitation is the potential loss of granularity, which can attenuate the contribution of small but clinically meaningful changes. This trade-off between fidelity to the raw signal and interpretability of the output is particularly relevant in critical care, where both precision and transparency are needed. DBNs, by supporting either discretized or continuous formulations, or hybrid models combining both, offer the methodological flexibility to adapt to the clinical context and the priorities of implementation. This ability to make temporal inferences, track clinical evolution, and derive predictive trajectories is what gives DBNs a great advantage in time series modeling in ICU settings.

### 3.4. Construction and Learning Methodology

For those convinced of the potential of such algorithmic tools, a natural question arises: how can a Bayesian network be constructed? The answer to this question rests on two fundamental elements: the structure of the graph (which nodes are connected and in which direction) and the probabilistic parameters (conditional probability tables, or CPTs). These two elements can be specified manually (based on medical expertise), learned automatically from data, or derived from a combination of both approaches. Before proceeding, certain prerequisites must be established. These include defining the target variable (future presence or absence of sepsis), specifying the available explanatory variables (e.g., temperature, leukocyte count, lactate level, ongoing treatments), determining the temporal framework (static or dynamic), and selecting the appropriate data granularity (discretized or continuous values, measurement frequency).

#### 3.4.1. Building the Network Structure

There are two distinct approaches for constructing the network structure: an expert approach (a priori structure) and an automated approach (learning structure).

**The expert approach** is carried out by clinicians and is based on medical knowledge of pathophysiology. The aim is to model the different variables based on scientifically proven cause-and-effect relationships. In the context of sepsis prediction, one can argue, that sepsis depends on the presence of an infection and the inflammatory response, while fever, leukocytosis, and hypotension depend on the host’s inflammatory response. This method has the advantage of transparency and causal control of the model, but it is also susceptible to cognitive biases and knowledge gaps.

**The structure learning approach** is based on artificial intelligence algorithms that determine the optimal network structure. These algorithms work either by maximizing a score (Hill Climbing, Tabu Search, etc.), by using conditional independence tests (PC Algorithm, FCI), or by using a hybrid approach combining the two previous methods (MMHC—Max-Min Hill Climbing) [19,20]. To put it simply, the algorithm examines the relationships between variables and automatically generates new connections. However, these automatically learned links do not necessarily imply a causal relationship; many may reflect mere statistical associations. For example, hyperleukocytosis may correlate with fever without being its direct cause. Therefore, it is essential to clinically validate each link to ensure that it reflects a plausible causal mechanism rather than a spurious correlation.

**Figure 5:**
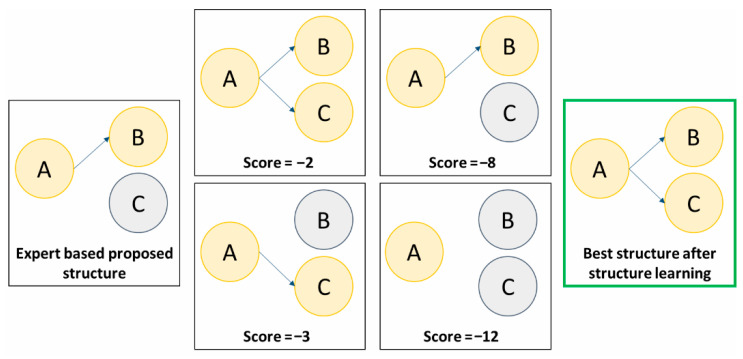
An example of Bayesian network structure learning based on hybrid method that combines expert knowledge with data-driven optimization. -**Expert-based proposed structure** (left panel): The initial network is defined according to domain expertise, with a directed edge from node A to node B, and variable C left unconnected. This reflects the expert’s prior belief about conditional dependencies. -**Data-driven candidate structures** (middle panels): Several alternative network structures are generated and scored based on a predefined scoring function (e.g., Bayesian Information Criterion, BIC). Each structure connects the variables A, B, and C differently. Lower scores indicate poorer fit to the data given the scoring criterion. -**Best structure after structure learning** (right panel): After evaluating all candidate structures, the optimal network which is the one with the highest score is selected. In this example, the best structure connects A → B and A → C, reflecting that A is a parent node influencing both B and C.

A third approach, complementary to the other two, has also emerged: the **hybrid approach**. Historically, artificial intelligence has distinguished between two main paradigms: symbolic methods and connectionist methods. Symbolic approaches, such as expert systems, rely on an explicit transcription of reasoning processes, generally designed by human experts. In contrast, connectionist approaches (data-driven learning) infer patterns exclusively from available data, without directly embedding prior knowledge about relationships. The hybrid approach bridges these two paradigms. First, a basic network structure is built using an expert-driven, symbolic process that models established scientific knowledge. This structure is then refined using a structure learning algorithm, which adds new relationships between variables by optimizing likelihood-based metrics to identify links not captured by existing knowledge (Figure 5). In doing so, it leverages the interpretability and domain grounding of symbolic models while benefiting from the adaptability and pattern-discovery capabilities of connectionist models.

This paradigm, increasingly referred to as neuro-symbolic AI, illustrates the ongoing convergence between symbolic and connectionist methods. Modern developments even allow prior knowledge to be incorporated into connectionist architectures through mechanisms such as transfer learning, graph neural networks, or guided reinforcement learning. In the medical domain, this hybrid strategy enables the combination of current clinical expertise with the discovery of non-obvious relationships between variables. However, expert validation remains essential to ensure that newly identified links are both clinically plausible and scientifically sound.

#### 3.4.2. Network Probability Learning

Once the structure has been defined, the parameters of the BN must be estimated, namely the conditional probability distributions (CPDs) associated with each node given its parents node. Just as a young doctor learns to make diagnoses, our Bayesian network will observe thousands of clinical cases from large hospital databases. It can learn to recognize patterns and adjust its beliefs by looking at either the frequency of observations (frequentist methods) or by considering the medical priors implemented in the network (Bayesian methods). The choice of method often depends on the amount of data available, the presence of prior knowledge about the parameters, and the tolerable computational complexity.

##### Frequentist Methods

Frequentist methods consider model parameters as fixed but unknown quantities. The objective is to estimate these values so that they “fit” the observed data as closely as possible. The most common method is Maximum Likelihood Estimation (MLE), which works by finding the parameter values that maximize the probability of observing the current data [21]. For each variable in the network, we consider all possible configurations of the parent variables on which it depends. For each of these combinations, we calculate the conditional probability of the target variable based on the frequency observed in the training data.

For example, supposing we want to model the probability of sepsis (target node) based on two parent clinical signs: the presence of fever and tachycardia. If, in a group of 20 patients with both fever and a heart rate above 100/min, 12 were found to be infected, then the estimated conditional probability of infection in this context is:

$$\hat{P}\left(sepsis\;=\;yes|\;fever\;=\;yes,\;HR>100\right)=\;12/20\;=\;0.6\;$$

All conditional probabilities associated with all combinations of parental variable values are then used to populate the probability table for the “infection” node. This intuitive and robust approach is the main method for estimating the parameters of a Bayesian network learned from complete data. This procedure, based solely on descriptive statistics from the training set, has the advantage of being transparent, explainable, and directly linked to the clinical experience collected. However, this method performs poorly on small data sets and if certain combinations of parent and child values never appear in the training data. This can be an issue during inference, as a zero probability can “block” the propagation of beliefs in the network. Finally, if the data is incomplete, direct MLE is no longer applicable. Iterative algorithms such as the Expectation-Maximization (EM) algorithm are then needed to estimate the parameters in the presence of missing data [22].

##### Bayesian Methods

In a Bayesian approach, the model parameters are considered to be uncertain rather than fixed. This uncertainty is modeled after assigning them a prior distribution (i.e., what is considered plausible before seeing the data). When clinical data are observed, they are used to update this initial belief by applying Bayes’ theorem. This gives us a new distribution, called the posterior distribution, which reflects what we think about the parameters after seeing the data. Bayesian methods are mainly robust to sparse data and frequent zeros because of the priors, which allow us to obtain more stable probabilities even with limited data. It is also possible to incorporate information on probability densities from experts in the field even before the data is observed (e.g., priors on the bacterial ecology of an ICU/ward for antibiotic resistance prediction tasks). However, for large networks, computational complexity can become an obstacle, requiring the use of sampling methods.

#### 3.4.3. Validation of the Learned Network

Once each BN has been constructed, it is essential to evaluate the quality of the prediction. This evaluation must be carried out through two complementary approaches:

- **Quantitative evaluation**: Often based on predictive performance metrics (AUROC, sensitivity, specificity, positive and negative predictive value, likelihood ratio and accuracy), but also on more global scores (Bayesian Information Criterion or Akaike Information Criterion). This analysis makes it possible to verify the clinical usefulness of the BN and compare it to other algorithms or scores available for the same prediction task.
- **Qualitative evaluation**: This is more a matter for the domain expert, who is responsible for verifying the pathophysiological consistency between the various relationships established by the network. This “expert-based” verification makes it possible to check that the structure learning has not implemented false relationships between variables (e.g., fever increases the leukocyte count).

As with any algorithm, best training practices recommend using a cohort to train the model and then validating it on an independent cohort. Comparing the algorithm with other state-of-the-art models (logistic regression, random forest, etc.) helps to consolidate its added value compared to existing literature.

## 4. Why Does Modeling Clinical Relationships Change the Game in Predicting Sepsis?

As we already saw, traditional AI models are mostly based on statistical correlations between variables, like “If CRP is high, it increases the risk of infection” or “If the patient has a fever and low blood pressure, it might be related to sepsis.” But in clinical practice, we don’t think exclusively in terms of correlations, but rather in terms of causal chains. We say to ourselves: “This pneumonia causes an inflammatory response that leads to hypotension but also hypoxemia due to alveolar filling with inflammatory cells.” We can visualize this causal chain in a BN, providing an almost educational touch for clinicians [15,23]. This ability to accurately track the chain of events that led to the prediction is a key factor in strengthening physicians’ confidence in the inferential capabilities of the tool. Health authorities (Food and Drug Administration—FDA, European Medicines Agency—EMA) also tend to favor transparent decision support systems, where the clinician retains control and understands the basis for the recommendation [24].

Sepsis is a condition in which we often make probabilistic decisions, whether about its occurrence, the pathogens involved, or antibiotic resistance. It is in this context that Bayesian networks come into their own in managing uncertainty. Where a conventional algorithm would force an opaque score (sepsis 80%) or a binary decision (sepsis vs. no sepsis), BNs produce an explicit conditional probability that is updated with each new piece of information, providing a confidence interval for the prediction (probability of sepsis between 80 and 95%). Uncertainty in the medical field should be weaved with the concept of epistemic humility. This entity refers to the explicit recognition of the limits of one’s knowledge and the acceptance of uncertainty as an intrinsic feature of reasoning and decision-making. Originally developed in the field of philosophy of science by *Zagzebski* in 1996, it emphasizes that models should not claim more certainty than the data allow, but instead provide transparent estimates of uncertainty [25,26]. BNs operationalize epistemic humility by explicitly quantifying uncertainty at each inference step. Rather than producing a categorical answer, they return probabilistic estimates that reflect both the available evidence and the residual uncertainty. In this sense, BNs align more closely with the way physicians reason under uncertainty, and may help foster trust by making explicit not only what is known, but also what remains unknown.

**Figure 6:**
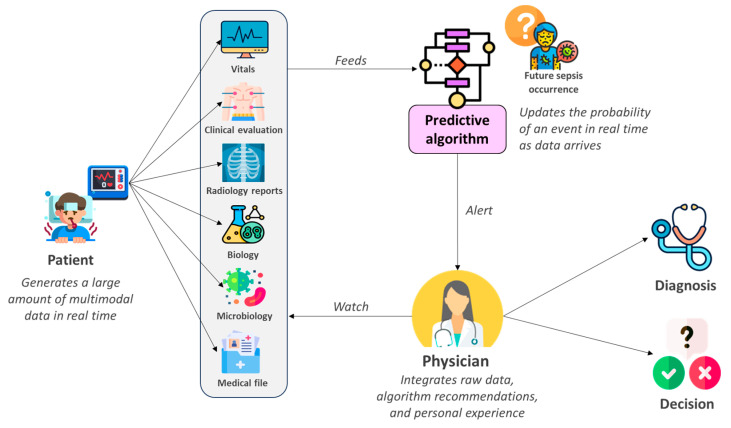
“*Human In The Loop*” pipeline for augmented medical decision process.

This approach is compatible with the Human in the Loop (HITL) paradigm, where humans remain in charge of the final decision and can choose to accept, reject, or modify the model’s recommendation without being relegated to the role of mere spectators [23,27,28]. This fruitful collaboration was demonstrated in a recent study in which radiologists were asked to interpret chest X-rays with the assistance of either a “black-box” model that provided only a raw prediction result, or the same model but with an explainability module that located the radiological lesions to explain the decision. Ultimately, doctors assisted by explainable AI achieved significantly better results [29]. In our case, the clinician’s involvement in developing the model structure, reinforced by continuous learning of the algorithm, is primarily aimed at consolidating a vision in which the machine assists humans rather than seeking to replace them (Figure 6).

## 5. Practical Applications of Bayesian Models in Predicting Sepsis

Here we present a literature review on the prediction of sepsis using artificial intelligence. A summary of these different studies is provided in Table 1.

### 5.1. Early Diagnosis Assistance

Several studies have shown that BNs are able to identify developing sepsis hours before conventional methods by using clinical data available upon admission (vital signs, laboratory tests, etc.) [30,31]. In 2020, Gupta et al. developed an automatic alert system based on a static BN algorithm of the Tree Augmented Naive Bayes (TAN) type [27]. The authors used automated techniques to select 13 variables, including clinical, biological, and demographic parameters. It is interesting to note that most of these variables are included in the SOFA score (used by the SEPSIS-III guidelines for the diagnosis of sepsis since 2016) and in the definition of SIRS. The final model achieved an Area Under Receiver Operating Curve (AUROC) of 0.84 for the prediction of sepsis, significantly outperforming the usual clinical scores in the same cohort (e.g., SIRS: 0.59; SOFA: 0.80). Notably, this high performance was achieved with fewer variables than other approaches, suggesting a tool that is both effective and easier to implement in practice. Another example is the SepsisFinder algorithm, trained to detect sepsis 48 h before its clinical onset [31]. This model is continuously updated (every hour) and has shown excellent performance (AUROC 0.95), outperforming the NEWS2 score used in standard triage (AUROC 0.87), while generating alerts on average 5.5 h earlier than the administration of antibiotics by the healthcare team. In comparison, a classic machine learning model (XGBoost) had a similar AUC but triggered its alerts later than the BN.

Some authors chose to use DBNs starting in 2012, using a combination of 11 variables collected upon admission to an emergency department. The algorithm was able to detect sepsis with an AUROC of 0.91 within the first 3 h of admission, rising to 0.94 after 24 h of observation. This temporal probabilistic model tracked the patient’s condition on an hourly basis and demonstrated that incorporating the dynamic evolution of vital signs over a few hours significantly improved the detection of early sepsis cases [30]. One of the main limitations of this study was the use of SIRS criteria, which have now been rendered obsolete by the latest recommendations, SEPSIS-III from 2016 [2].

The application of these BN methods to the early detection of sepsis by exploiting initial clinical data (vital signs, routine laboratory tests, and sometimes free-text notes) offers a promising alternative to traditional frequentist algorithms. The explicit inclusion of the temporal dimension via DBNs is a key success factor, enabling the model to gain sensitivity in detecting subtle abnormal trends that are precursors to sepsis. Projects such as InSight [32] in sepsis prediction or PREDICT [33] in VAP prediction have shown that integrating this temporal dynamics into other types of algorithms offers excellent inference capabilities (AUROC > 0.9). However, the main limitation to the routine implementation of such tools is the automated retrieval of the variables necessary for the algorithm to function properly by connecting them to the hospital information system (HIS). Otherwise, clinicians would have to spend a considerable amount of time manually collating the variables, which would be extremely time-consuming and ultimately counterproductive.

### 5.2. Assistance with Sorting and Prognosis

Once sepsis has been identified or suspected, it is crucial to assess its severity and prognosis in order to guide the patient’s referral within the healthcare system (admission to intensive care vs. conventional ward) and anticipate the necessary resources. In this context, BNs have been used to estimate the risk of unfavorable outcomes (septic shock, multiple organ failure, death) by combining multiple factors (vital signs, biological data, medical history, etc.) in a probabilistic manner [34].

In 2018, Wang et al. derived a DBN model to predict the risk of mortality in sepsis patients in intensive care [34]. The development of their model was founded on an expert structure established by physicians, integrating both structured physiological variables and unstructured textual information from the medical records. The model demonstrated remarkable prognostic capabilities, with an AUROC of 0.91, surpassing conventional prognostic scores. Furthermore, the reclassification analysis conducted by the authors corroborates the hypothesis that the BN model provides enhanced discrimination relative to these scores. In practice, the implementation of such a tool could facilitate the early identification of sepsis patients who are at high risk of mortality, thereby enabling the delivery of targeted monitoring and intensive care. This work illustrates a hybrid approach that combines clinical expertise and machine learning to enhance prognosis accuracy.

BNs can also be used to model the progression of organ failure in sepsis. This was the approach taken by Peelen et al. in 2010, using a hierarchical DBN in intensive care patients with sepsis [35]. The objective was twofold: first, to identify temporal patterns of organ dysfunction (i.e., to predict the sequence in which organs will deteriorate), and second, to virtually test clinical hypotheses about the progression of sepsis. The model can predict the future progression of the disease and provide decision-making guidance in intensive care (e.g., alerting clinicians that a patient is at risk of developing renal failure within 24 h). This approach goes beyond simple mortality prediction to provide a more detailed view of future clinical conditions, guiding clinicians in proactively choosing interventions. Here, we see the potential of BNs for patient triage and prognosis. Implementing this type of model in an emergency department, for example, would provide clinicians with valuable insights, helping them to prevent life-threatening misdiagnosis.

### 5.3. Assistance with Treatment Strategy

The use of BNs to guide real-time therapeutic strategy remains an emerging field. However, several factors suggest that they can provide significant added value in clinical decision-making for the treatment of sepsis. The term “treatment strategy” covers two aspects here: the rapid initiation of proven treatments (antibiotics, fluid resuscitation, vasopressors, etc.) as soon as sepsis is suspected, and the personalized selection or adjustment of interventions based on the patient’s profile and progression.

One of the pioneering works in this field is the TREAT project, which was drawn up in 2007 [36]. This ambitious project aimed to create a clinical decision support system (CDSS) designed to improve antibiotic treatment in hospitalized patients with common bacterial infections. The system was based on a causal probabilistic network designed to model relationships between signs, symptoms of infection, bacteria, and antibiotics. This model, tested in a multicenter cluster randomized controlled trial involving 2326 patients, recommended appropriate antibiotic treatment for 70% of patients, compared with 57% actually prescribed by physicians (*p* = 0.0001). The rate of appropriate empirical antibiotic treatment was 73% in the intervention wards compared with 64% in the control wards (adjusted OR 1.48). For patients treated according to the first three TREAT recommendations, the rate of appropriate treatment reached 85%. The cost of antibiotics prescribed by TREAT was nearly 50% lower than those prescribed by physicians, with a corresponding decrease in the use of broad-spectrum antibiotics. The use of the tool also shortened the length of hospital stays. This system was also remarkably complex, with the ability to predict 22 different pathogens or groups of pathogens, which would be almost impossible with traditional statistical models due to the amount of data required.

Modeling the cause-and-effect relationships between different interventions makes it possible to guide the choice or customization of therapies beyond antibiotic therapy alone. These algorithms can theoretically serve as a simulator to compare different treatment scenarios estimating how specific therapeutic actions might influence patient outcomes [37]. For example, the model could predict the probability of blood pressure stabilization following the administration of an additional fluid bolus, or anticipate the hemodynamic effects of initiating a vasopressor. By integrating the patient’s current data, the network continuously updates these probabilities in real time, enabling dynamic evaluation of different treatment strategies.

In current clinical practice, the direct integration of a BN to recommend a specific treatment remains uncommon. However, by facilitating continuous risk stratification, these models are already influencing strategy: for example, by flagging a patient as high risk, they encourage the team to apply all prompt intensive measures (early central catheter placement, begging volume expansion early, non-invasive ventilation, etc.), while a patient flagged as low risk could avoid unnecessary invasive procedures. Overall, the contribution of Bayesian networks to therapeutic strategy is mainly reflected in the optimization of therapeutic timing and intensity and in the potential for personalized care guided by the probability of success of each intervention. However, despite promising results, few of these models have been translated into operational clinical tools. There are many reasons for this: the need for multicenter validation, integration into hospital information system, but above all, clinician buy-in. It is therefore essential that future studies involve healthcare professionals from the design stage of the model, in line with the Human-in-the-Loop approach.

**Table 1:** Summary of Key Studies on Sepsis Prediction Using Artificial Intelligence.

Study	Prediction Outcome	Algorithms	Main Results	Population	Prediction Horizon	Data Used in Algorithm
*Nachimuthu & Haug, 2012* * [30]*	**Sepsis:** SIRS (at least 2 criterions) + suspected infection	Dynamic Bayesian Networks (DBN)	**DBN**: AUROC: 0.94 Se 0.86; Sp 0.95	ER patients; *n* = 741 patients.	Prediction of sepsis within the first 24 h	WBC, immature neutrophil %, HR, MAP, DBP, SBP, temperature, RR, PaCO_2_, age
*Henry* et al.,* 2015* *[14]*	**Septic shock**: Sepsis (SIRS + suspected infection) + hypotension after ≥20 mL/kg fluid resuscitation in past 24 h	**TREWScore**: Cox model **Comparators**: SIRS, MEWS	**TREWScore**: AUROC: 0.83 Sp 0.67; Se 0.85 **SIRS**: Se 0.74; Sp 0.64	MIMIC-II database. *n* = 16,234 patients.	Real-time risk update with no fixed window	GCS, platelets, BUN/creatinine ratio, arterial pH, temperature, RR, WBC, bicarbonate, HR/SBP (shock index), SBP, HR
*Haug et Ferraro, 2016* * [38]*	**Sepsis**: Retrospective identification by ICD codes in EHR.	- Bayesian Network (BN) - Tree-Augmented Naïve Bayesian Network (TAN)	**BN**: AUROC: 0.97 Se 0.60; PPV: 0.38 **TAN**: AUROC: 0.97 Se 0.60; PPV: 0.37	ER patients. *n* = 186,725 patients	Prediction using data from first 90 min	Age, MAP, temperature, HR, WBC
*Desautels* et al.,* 2016* *[32]*	**Sepsis**: SEPSIS-III criteria	**Insight** (Gradient Tree Boosting) **Comparators**: SOFA, qSOFA, MEWS, SIRS	**Insight**: AUROC: 0.74 Se 0.80; Sp 0.54 **SOFA**: AUROC: 0.73 Se 0.80; Sp 0.48	MIMIC-III dataset. *n* = 22,853 ICU stays.	Sepsis prediction 4 h in advance using 2-h window	Age, RR, DBP, SBP, temperature, SaO_2_, GCS
*Barton* et al.,* 2019* *[39]*	**Sepsis**: SEPSIS-III criteria	**XGBoost** **Comparators**: SOFA, qSOFA, MEWS, SIRS	**XGBoost**: AUROC: 0.84 Se 0.80; Sp 0.72 **SOFA**: AUROC: 0.72 Se 0.78; Sp 0.59	MIMIC-III *n* = 21,507 patients	Sepsis prediction up to 24 h in advance	SaO_2_, HR, SBP, DBP, temperature, RR
*Gupta* et al.,* 2020* *[27]*	**Sepsis:** SEPSIS-III criteria Diagnosis inferred via mortality	**Tree-Augmented Naïve Bayesian Network** (TAN) **Comparators**: SIRS, qSOFA, MEWS, SOFA scores	**TAN**: AUROC: 0.84 Se 0.71; Sp 0.80 **SOFA**: AUROC: 0.80 Se 0.71; Sp 0.75	Cerner Corporations HIPAA-compliant Health Facts database *n* = 63 million patients	Real-time sepsis risk update as data arrives	SBP, GCS, RR, WBC, creatinine, PaO_2_/FiO_2_
*Shashikumar* et al.,* 2021* *[40]*	**Sepsis**: SEPSIS-III criteria	Deep Artificial Intelligence Sepsis Expert (DeepAISE): Gated Recurrent Unit multi-layer deep network	**DeepAISE:** AUROC: 0.90 Se 0.85; Sp 0.80	ICU patients. *n* = 25,820 patients	Sepsis prediction 4 h in advance	65 features incl. MAP, HR, SpO_2_, SBP, DBP, RR, GCS, PaO_2_, FiO_2_, WBC, creatinine, BUN, lactate, CRP, demographics
*Valik* et al.,* 2023* *[31]*	**Sepsis**: SEPSIS-III criteria	**SepsisFinder** (CPN) **Comparators**: LightGBM, NEWS2	**SepsisFinder**: AUROC: 0.95 Se 0.85; Sp 0.97 **LightGBM**: AUROC: 0.949	Non-ICU hospital admissions *n* = 55,655	Sepsis prediction within 48 h	HR, MAP, RR, SpO_2_, O_2_ flow, GCS, CRP, WBC, platelets, bilirubin, creatinine, BUN, albumin, lactate, HCO_3_, pH, department, time since surgery

Glasgow Coma Scale (GCS), Blood Urea Nitrogen (BUN), Respiratory Rate (RR), White Blood Cell count (WBC), Systolic Blood Pressure (SBP), Heart Rate (HR), Mean Arterial Pressure (MAP), Diastolic Blood Pressure (DBP), Area Under Receiver Operating Curve (AUROC), Sensitivity (Se), Specificity (Sp), Systemic Inflammatory Response Syndrome (SIRS), Electronic Health Records (EHR), Temperature (T°), Partial pressure of arterial carbon dioxide (PaCO_2_), Partial pressure of arterial oxygen/fraction of inspired oxygen (PaO_2_/FiO_2_), Arterial pH (pH), C-reactive protein (CRP), Oxygen saturation (SaO_2_/SpO_2_), Oxygen flow rate (O_2_ flow6. Future prospects and clinical integration.

The various areas of application for BNs described above suggest a wide range of potential future uses.

ICUs naturally generate a continuous flow of multimodal data (cardiovascular monitoring, laboratory results, medical record text, etc.). These units would therefore provide an excellent jumping-off point for implementing such tools. A promising approach would be to implement Bayesian networks able to update themselves in real time based on these data streams. The “sequential” arrival of data (not all the data required by the algorithm is available at the same time) limits the practical use of traditional algorithms, which require a complete data vector to make their predictions. BNs correct this pitfall by naturally managing missing data. Technically, this involves optimizing propagation algorithms (to instantly provide new probabilities each time data is received) and possibly using online learning approaches to update certain BN parameters over time if new trends emerge (while avoiding degrading the model with noise). This real-time integration goes hand in hand with the architecture of electronic health records and monitoring systems. In addition, the proliferation of health data into “big health data,” particularly with the rise of health data warehouses, could feed very large-scale BNs to refine a priori probabilities or relationships between comorbidities and sepsis. We can imagine “global” BNs trained on millions of cases that would provide robust inference data that could then be adapted to each care unit.

Beyond their role as predictive models, BNs also provide a formal framework for causal inference. While prediction aims to estimate the likelihood of an event given observed data, causal inference addresses a different question: *What would happen if we were to intervene?* BNs are able to deep dive into cause inference permitting interventions simulations by actively modifying the state of a variable and propagating the consequences throughout the network [41]. This capacity to move from association to causation opens the possibility of evaluating alternative treatment strategies, personalizing interventions, and designing decision-support systems that go beyond risk stratification to provide actionable recommendations. However, such applications require careful expert validation and robust data, as spurious or mis-specified causal links could mislead clinical decision-making.

Bayesian modeling of dynamic variables helps to overcome the main limitations of static networks. Among the various Bayesian configurations explored in the sepsis prediction literature, DBNs have consistently shown the best performance. Incorporating temporal information almost recurrently improves predictive accuracy compared to static models [30,33,34]. In addition, integrating hidden variables representing the patient’s latent physiological state will ultimately make it possible to better filter out random fluctuations and detect the signal of early sepsis amid the noise of physiological variations. A promising direction is therefore to combine static and dynamic structures, leveraging the strengths of both approaches to enhance predictive power. The interest of DBNs extends beyond sepsis, as longitudinal data are abundant in intensive care and particularly relevant for forecasting outcomes such as acute kidney injury or impending hypotension. However, several questions remain unresolved regarding their optimal use. One concerns whether to use raw measurements directly, or to apply feature engineering strategies (e.g., rolling averages, percentage changes) to extract more informative temporal signals. Another issue relates to the handling of continuous variables: while classical BN implementations often rely on discretization, which may result in some information loss, DBNs can also be implemented as hybrid models using conditional linear Gaussian distributions, thereby allowing raw values to be incorporated directly [42]. Finally, the question of the temporal window to be modeled also remains open: should inference be based on only the previous value, the last two or three values, or rather on the estimated rate of change? It is likely that the speed of variation is the most clinically relevant parameter, yet no formal recommendations currently exist regarding the optimal DBN configuration for ICU data.

Furthermore, despite all their advantages, DBNs are not as powerful in modeling dynamic variables as other AI techniques such as deep neural networks. It is therefore increasingly feasible to combine BNs with other AI techniques to take advantage of the benefits of each. We can imagine using deep Bayesian networks, which incorporate neural networks within a Bayesian framework. The idea would be, for example, to create a node whose conditional probability is provided by a small neural network trained on complex data such as images or text. This could make it possible to process unstructured data (imaging, clinical notes) while retaining the interpretable overall architecture of the BN [34].

From a regulatory standpoint, AI-based clinical decision support software is receiving increasing attention from official authorities (FDA, EMA, etc.). A Bayesian network used as a medical device software must meet criteria for safety, reliability, and traceability. The FDA has issued guidelines on CDSSs (Clinical Decision Support Systems) that distinguish between algorithms that “only” provide information to clinicians (not subject to approval) and those that directly suggest an action or diagnosis (often subject to approval) [43]. In the case of sepsis, a tool that alerts “This patient is likely to have sepsis, treat now” is close to a diagnosis and could be regulated. Fortunately, BNs benefit from their transparency: it is easier to “open the hood” to check why the algorithm makes this statement which may facilitate regulatory acceptance. It is also possible to formally verify certain properties of the network (for example, ensuring that in the absence of any signs, the probability of sepsis remains low), which in a neural network must be tested empirically. Close collaboration between developers, clinicians, and authorities will therefore be essential to certify these models and facilitate their implementation.

Beyond simple regulation, these XAI issues also raise the question of the medical-legal responsibility of doctors who use algorithmic assistance. Who could be sued when something goes wrong? The physician who followed the algorithm’s prediction? The algorithm’s designer? In practice, it is the clinician who will be held liable, unless they can prove that they acted in accordance with best practice recommendations [44]. Consequently, the use of an algorithm in clinical practice appears to be “stillborn” if it is unable to provide at least some of the reasons behind its prediction. This approach reinforces the Human In The Loop vision, whereby the human clinician remains in control of these decisions in all cases and uses AI not as a substitute but as a technological aid.

Of course, adopting these techniques requires an essential step, namely conducting prospective clinical studies to assess the impact of these algorithms in practice. This type of study would also enable user feedback to be collected in order to refine the interface: how can the results of the BN be best explained to clinicians? What form of visualization (interactive graph, display of contributing factors, etc.) is most convincing to them? The HITL vision encourages us to consider the result of BN inference not as an absolute truth but rather as a digital biomarker. The major advantage of such a tool is that it synthesizes a large number of clinical and biological variables into an interpretable signal, particularly in an environment where clinicians are constantly exposed to information overload. A critical question, however, concerns the threshold at which such probabilistic outputs should trigger clinical action. This issue is not purely statistical but involves ethical and contextual considerations, and may depend on consensus within each ICU rather than the judgment of an individual clinician. Addressing this challenge requires dedicated studies to determine how probabilistic estimates can be operationalized into actionable thresholds that maximize patient benefit while minimizing risks of over- or under-treatment. Future studies will also need to evaluate how the use of a BN fits into the workflow without increasing the cognitive load or generating excessive alerts.

Constant advances in medical computing are making it possible to deploy BNs directly in hospital systems. Today, a standard server can perform Bayesian inference for hundreds of patients in parallel in near real time. More complex deep learning algorithms, on the other hand, would quickly be limited by the available computing power, which restricts their scope of application. Integration with electronic health records via standard medical data communication protocols (HL7, FHIR, etc.) would make it possible to automatically retrieve the necessary information (vital signs, tests) and return the probability calculation in the form of an alert in the patient’s electronic health record. However, until these models are seamlessly integrated into our business software (electronical patient records, monitoring), they will remain at the proof-of-concept stage. Their real value will depend on their ability to fit into existing workflows without replacing them.

Finally, beyond predictive performance, the clinical evaluation of AI models requires a multidimensional approach. While metrics such as AUROC, sensitivity, and specificity are necessary to benchmark raw predictive accuracy, they are insufficient on their own to justify adoption at the bedside. Clinicians also need to assess other critical dimensions: the model’s robustness to missing or noisy data (a common situation in the ICU), its interpretability and ability to provide stable explanations, its capacity to quantify uncertainty rather than deliver categorical answers, and its alignment with established medical reasoning. Workflow integration and usability within existing electronic health record systems are equally essential. Ultimately, the most decisive criterion is prospective validation and proven impact on patient outcomes, as illustrated by the TREWS trial, where an explainable sepsis early warning system led to a significant reduction in mortality. A structured framework for evaluation (Table 2) may therefore help clinicians compare the many models currently being proposed and identify those most likely to deliver meaningful clinical value.

## 6. Conclusions

Bayesian networks offer significant advantages in applications such as sepsis prediction. Through conditional modeling of variables, management of diagnostic uncertainty, and handling of missing data, these algorithms represent serious contenders compared to traditional approaches in supporting clinicians in this difficult diagnosis. The wide range of uses, from early diagnosis to prognosis estimation and potentially treatment optimization, offers many prospects for future applications. The major advantage of BNs over more traditional algorithms is undoubtedly the interpretability of their predictions, which would greatly facilitate their informed use by the medical community.

Future prospects are exciting: with the accumulation of big health data and the growing need for real-time support tools, we can expect BNs (possibly combined with other AI techniques) to become standard components of clinical monitoring systems. To achieve this, we will need to define a multidisciplinary approach (clinicians, data scientists, computer scientists) in order to overcome implementation and reliability challenges. The challenge is not only to build high-performance models, but also to develop tools that are clinically acceptable, transparent, and flexible enough to adapt to the daily reality of physicians. It is important to emphasize, however, that alternative algorithmic approaches should not be disregarded. Certain predictive tasks can only achieve high levels of performance through frequentist or data-driven algorithms, particularly in fields such as image analysis, complex time-series modeling (e.g., EEG, ECG), or natural language processing. The larger the volume of training data, the more powerful and precise these models become in their inferences. In the current era of medical big data, it would be unrealistic to sacrifice predictive performance solely for the sake of explainability. Nevertheless, given that most physicians remain relatively untrained in the bedside use of AI, the intrinsic interpretability and native transparency of Bayesian networks may represent a pragmatic entry point to foster greater clinical acceptance of these tools, which are still often met with skepticism. This reluctance could be mitigated further by the possibility of incorporating expert knowledge into Bayesian structures, thereby aligning clinicians’ theoretical understanding with the learning capabilities of machine algorithms. Nevertheless, we must keep in mind that all these models are not intended to replace the clinician’s knowledge and experience, but rather to reinforce it. In a context as uncertain, urgent, and multidimensional as sepsis diagnosis, this type of AI is not a technical option: it is a clinical necessity.

## Figures and Tables

**Table 2:** Proposed framework for evaluating AI models in sepsis prediction.

Dimension	Typical Metric/Approach	Relevance for Clinical Adoption
**Predictive performance**	AUROC, AUPRC, sensitivity, specificity, calibration curves	Necessary to establish **baseline model** **accuracy**, but not sufficient for bedside use
**Robustness to missing or noisy data**	Performance under simulated missingness, imputation strategies, sensitivity analyses	Critical in ICU **where incomplete data is common** (labs not yet available)
**Explainability/Interpretability**	Intrinsic model transparency (BN), post-hoc methods (SHAP, LIME)	Determines whether clinicians can **understand** and **trust the recommendation**
**Uncertainty quantification**	Confidence intervals, posterior distributions, predictive intervals	Helps **avoid over-reliance on predictions** and supports **cautious decision-making**
**Alignment with clinical reasoning**	Face validity, concordance with known causal relationships	**Facilitates clinician acceptance** and integration into decision-making processes
**Workflow integration**	Real-time capability, compatibility with EHR systems, usability testing	Ensures **feasibility** of **deployment** in high-intensity ICU settings
**Impact on outcomes**	Prospective validation, randomized controlled trials.	Ultimately, the only measure that **justifies clinical adoption**

## Data Availability

No new data were created or analyzed in this study.

## Abbreviations

The following abbreviations are used in this manuscript:

AI	Artificial Intelligence
AUROC	Area Under the Receiver Operating Characteristic Curve
BN	Bayesian Network
BUN	Blood Urea Nitrogen
CRP	C-Reactive Protein
CPT	Conditional Probability Table
DBN	Dynamic Bayesian Network
DBP	Diastolic Blood Pressure
EHR	Electronic Health Record
FiO_2_	Fraction of Inspired Oxygen
GCS	Glasgow Coma Scale
HR	Heart Rate
MAP	Mean Arterial Pressure
MEWS	Modified Early Warning Score
MIMIC	Medical Information Mart for Intensive Care
O_2_ flow	Oxygen Flow Rate
PaCO_2_	Partial Pressure of Arterial Carbon Dioxide
PaO_2_/FiO_2_	Partial Pressure of Arterial Oxygen/Fraction of Inspired Oxygen
pH	Arterial pH
PPV	Positive Predictive Value
qSOFA	quick Sequential Organ Failure Assessment
RR	Respiratory Rate
SaO_2_	Arterial Oxygen Saturation
SBP	Systolic Blood Pressure
Se	Sensitivity
SIRS	Systemic Inflammatory Response Syndrome
SOFA	Sequential Organ Failure Assessment
Sp	Specificity
SpO_2_	Peripheral Oxygen Saturation
TAN	Tree-Augmented Naive Bayes
T°	Temperature
WBC	White Blood Cell count
xAI	Explainable Artificial Intelligence

## References

[1] Rudd K.E., Johnson S.C., Agesa K.M., Shackelford K.A., Tsoi D., Kievlan D.R., Colombara D.V., Ikuta K.S., Kissoon N., Finfer S. (2020). Global, regional, and national sepsis incidence and mortality, 1990–2017: Analysis for the Global Burden of Disease Study. Lancet.

[2] Singer M., Deutschman C.S., Seymour C.W., Shankar-Hari M., Annane D., Bauer M., Bellomo R., Bernard G.R., Chiche J.D., Coopersmith C.M. (2016). The Third International Consensus Definitions for Sepsis and Septic Shock (Sepsis-3). JAMA.

[3] Evans L., Rhodes A., Alhazzani W., Antonelli M., Coopersmith C.M., French C., Machado F.R., Mcintyre L., Ostermann M., Prescott H.C. (2021). Surviving sepsis campaign: International guidelines for management of sepsis and septic shock 2021. Crit. Care Med..

[4] Seymour C.W., Gesten F., Prescott H.C., Friedrich M.E., Iwashyna T.J., Phillips G.S., Lemeshow S., Osborn T., Terry K.M., Levy M.M. (2017). Time to Treatment and Mortality during Mandated Emergency Care for Sepsis. N. Engl. J. Med..

[5] Liu V.X., Fielding-Singh V., Greene J.D., Baker J.M., Iwashyna T.J., Bhattacharya J., Escobar G.J. (2017). The Timing of Early Antibiotics and Hospital Mortality in Sepsis. Am. J. Respir. Crit. Care Med..

[6] Isaranuwatchai S., Buppanharun J., Thongbun T., Thavornwattana K., Harnphadungkit M., Siripongboonsitti T. (2025). Early antibiotics administration reduces mortality in sepsis patients in tertiary care hospital. BMC Infect. Dis..

[7] Adams R., Henry K.E., Sridharan A., Soleimani H., Zhan A., Rawat N., Johnson L., Hager D.N., Cosgrove S.E., Markowski A. (2022). Prospective, multi-site study of patient outcomes after implementation of the TREWS machine learning-based early warning system for sepsis. Nat. Med..

[8] Lauritsen S.M., Kalør M.E., Kongsgaard E.L., Lauritsen K.M., Jørgensen M.J., Lange J., Thiesson B. (2020). Early detection of sepsis utilizing deep learning on electronic health record event sequences. Artif. Intell. Med..

[9] Van de Sande D., van Genderen M.E., Huiskens J., Gommers D., van Bommel J. (2021). Moving from bytes to bedside: A systematic review on the use of artificial intelligence in the intensive care unit. Intensive Care Med..

[10] Guidotti R., Monreale A., Ruggieri S., Turini F., Giannotti F., Pedreschi D. (2019). A Survey of Methods for Explaining Black Box Models. ACM Comput. Surv..

[11] Champendal M., Müller H., Prior J.O., dos Reis C.S. (2023). A scoping review of interpretability and explainability concerning artificial intelligence methods in medical imaging. Eur. J. Radiol..

[12] Goh G.S.W., Lapuschkin S., Weber L., Samek W., Binder A. Understanding Integrated Gradients with SmoothTaylor for Deep Neural Network Attribution. Proceedings of the 2020 25th International Conference on Pattern Recognition (ICPR).

[13] Gavito A.T., Klabjan D., Utke J. (2024). Multi-Layer Attention-Based Explainability via Transformers for Tabular Data. arXiv.

[14] Henry K.E., Hager D.N., Pronovost P.J., Saria S. (2015). A targeted real-time early warning score (TREWScore) for septic shock. Sci. Transl. Med..

[15] Polotskaya K., Muñoz-Valencia C.S., Rabasa A., Quesada-Rico J.A., Orozco-Beltrán D., Barber X. (2024). Bayesian Networks for the Diagnosis and Prognosis of Diseases: A Scoping Review. Mach. Learn. Knowl. Extr..

[16] Pearl J. (1995). From Bayesian Networks to Causal Networks. Mathematical Models for Handling Partial Knowledge in Artificial Intelligence.

[17] Russell S.J., Norvig P., Davis E. (2010). Artificial Intelligence: A Modern Approach.

[18] Schupbach J., Pryor E., Webster K., Sheppard J. Combining Dynamic Bayesian Networks and Continuous Time Bayesian Networks for Diagnostic and Prognostic Modeling. Proceedings of the 2022 IEEE AUTOTESTCON.

[19] Tsamardinos I., Brown L.E., Aliferis C.F. (2006). The max-min hill-climbing Bayesian network structure learning algorithm. Mach. Learn..

[20] Scutari M., Graafland C.E., Gutiérrez J.M. (2019). Who Learns Better Bayesian Network Structures: Accuracy and Speed of Structure Learning Algorithms. arXiv.

[21] Kochenderfer M.J., Wheeler T.A., Wray K.H. (2022). Algorithms for Decision Making.

[22] Ben F., Kalti K., Ali M. (2011). The threshold EM algorithm for parameter learning in bayesian network with incomplete data. Int. J. Adv. Comput. Sci. Appl..

[23] Nicora G., Catalano M., Bortolotto C., Achilli M.F., Messana G., Lo Tito A., Consonni A., Cutti S., Comotto F., Stella G.M. (2024). Bayesian Networks in the Management of Hospital Admissions: A Comparison between Explainable AI and Black Box AI during the Pandemic. J. Imaging.

[24] (2025). Health C for D and R. Artificial Intelligence in Software as a Medical Device. FDA. https://www.fda.gov/medical-devices/software-medical-device-samd/artificial-intelligence-software-medical-device.

[25] Zagzebski L.T. (1996). Virtues of the Mind: An Inquiry into the Nature of Virtue and the Ethical Foundations of Knowledge.

[26] Kidd I.J. (2016). Intellectual Humility, Confidence, and Argumentation. Topoi.

[27] Gupta A., Liu T., Shepherd S. (2020). Clinical decision support system to assess the risk of sepsis using Tree Augmented Bayesian networks and electronic medical record data. Health Inform. J..

[28] Zhang S., Yu J., Xu X., Yin C., Lu Y., Yao B., Tory M., Padilla L.M., Caterino J., Zhang P. Rethinking Human-AI Collaboration in Complex Medical Decision Making: A Case Study in Sepsis Diagnosis. Proceedings of the 2024 CHI Conference on Human Factors in Computing System.

[29] Senoner J., Schallmoser S., Kratzwald B., Feuerriegel S., Netland T. (2024). Explainable AI improves task performance in human-AI collaboration. arXiv.

[30] Nachimuthu S.K., Haug P.J. (2012). Early detection of sepsis in the emergency department using Dynamic Bayesian Networks. AMIA Annu. Symp. Proc..

[31] Valik J.K., Ward L., Tanushi H., Johansson A.F., Färnert A., Mogensen M.L., Pickering B.W., Herasevich V., Dalianis H., Henriksson A. (2023). Predicting sepsis onset using a machine learned causal probabilistic network algorithm based on electronic health records data. Sci. Rep..

[32] Desautels T., Calvert J., Hoffman J., Jay M., Kerem Y., Shieh L., Shimabukuro D., Chettipally U., Feldman M.D., Barton C. (2016). Prediction of Sepsis in the Intensive Care Unit With Minimal Electronic Health Record Data: A Machine Learning Approach. JMIR Med. Inform..

[33] Agard G., Roman C., Guervilly C., Forel J.-M., Orléans V., Barrau D., Auquier P., Ouladsine M., Boyer L., Hraiech S. (2025). An Innovative Deep Learning Approach for Ventilator-Associated Pneumonia (VAP) Prediction in Intensive Care Units—Pneumonia Risk Evaluation and Diagnostic Intelligence via Computational Technology (PREDICT). J. Clin. Med..

[34] Wang T., Velez T., Apostolova E., Tschampel T., Ngo T.L., Hardison J. (2018). Semantically Enhanced Dynamic Bayesian Network for Detecting Sepsis Mortality Risk in ICU Patients with Infection. arXiv.

[35] Peelen L., de Keizer N.F., de Jonge E., Bosman R.-J., Abu-Hanna A., Peek N. (2010). Using hierarchical dynamic Bayesian networks to investigate dynamics of organ failure in patients in the Intensive Care Unit. J. Biomed. Inform..

[36] Leibovici L., Paul M., Nielsen A.D., Tacconelli E., Andreassen S. (2007). The TREAT project: Decision support and prediction using causal probabilistic networks. Int. J. Antimicrob. Agents.

[37] Petrungaro B., Kitson N.K. (2025). Constantinou AC. Investigating potential causes of Sepsis with Bayesian network structure learning. arXiv.

[38] Haug P., Ferraro J. Using a Semi-Automated Modeling Environment to Construct a Bayesian, Sepsis Diagnostic System. Proceedings of the 7th ACM International Conference on Bioinformatics, Computational Biology, and Health Informatics.

[39] Barton C., Chettipally U., Zhou Y., Jiang Z., Lynn-Palevsky A., Le S., Calvert J., Das R. (2019). Evaluation of a machine learning algorithm for up to 48-hour advance prediction of sepsis using six vital signs. Comput. Biol. Med..

[40] Shashikumar S.P., Josef C.S., Sharma A., Nemati S. (2021). DeepAISE—An interpretable and recurrent neural survival model for early prediction of sepsis. Artif. Intell. Med..

[41] Li F., Ding P., Mealli F. (2023). Bayesian causal inference: A critical review. Philos. Trans. R. Soc. A.

[42] Kungurtsev V., Idlahcen F., Rysavy P., Rytir P., Wodecki A. (2024). Learning Dynamic Bayesian Networks from Data: Foundations, First Principles and Numerical Comparisons. arXiv.

[43] U.S. Food & Drug Administration (2022). Clinical Decision Support Software—Guidance for Industry and Food and Drug Administration Staff.

[44] Mello M.M., Guha N. (2024). Understanding Liability Risk from Using Health Care Artificial Intelligence Tools. N. Engl. J. Med..

